# Supplementary material for: Prolonged Survival after Recurrence in HCC Resected Patients Using Repeated Curative Therapies: Never Give Up!
Source: Cancers (Basel). 2022 Dec 30;15(1):232. doi: 10.3390/cancers15010232 (PMC9818493; doi:10.3390/cancers15010232)
Supplement: Supplementary file 1 [file cancers-15-00232-s001.zip › cancers-2031110-supplementary.pdf]

cancers

MDPI

Supplementary Materials

Prolonged Survival after Recurrence in HCC Resected Patients Using Repeated Curative Therapies: Never Give Up!

Cyprien Toubert, Boris Guiu, Bader Al Taweel, Eric Assenat, Fabrizio Panaro, François-Regis Souche, Jose Ursic-Bedoya, Francis Navarro and Astrid Herrero

Table S1. cut-off of early versus late recurrence using "minimum-*p*-value" method.

Cut-Off (Months After Initial Surgery)	Early Recurrence Group		Late Recurrence Group	
	Median SAR	Median SAR difference	Median SAR	*p*-value for SAR
1	3	24	27	0.285
3	13	16	29	0.004
5	18	14	32	0.006
7	17	18	35	0.003
9	17	21	38	0.007
11	17	23	40	0.003
13	18	22	40	0.004
15	19	21	40	0.011
17	19	21	40	0.009
19	22	14	36	0.094
21	23	0	23	0.198
23	23	11	34	0.445

SAR: Survival after recurrence .
